# Chemistry, Bioactivity, and Prediction of the Quality Marker (Q-Marker) of *Ferula* Plants in China: A Review

**DOI:** 10.3390/molecules28135191

**Published:** 2023-07-04

**Authors:** Yerlan Bahetjan, Muguli Muhaxi, Kejian Pang, Murat Kizaibek, Hui Tang, Fatemeh Sefidkon, Xinzhou Yang

**Affiliations:** 1 International Cooperation Base for Active Substances in Traditional Chinese Medicine in Hubei Province, School of Pharmaceutical Sciences, South-Central Minzu University, 182 Min-Zu Road, Wuhan 430074, China; yerlansb@163.com; 2 College of Biological and Geographical Sciences, Yili Normal University, Yining 835000, China; muguli@163.com (M.M.); arnebia@126.com (K.P.); 3 Traditional Kazakh Medicine Research Institute of Ili Kazakh Autonomous Prefecture, Yining 835000, China; murat_kizaibek@sina.com; 4 Key Laboratory of Xinjiang Phytomedicine Resource and Utilization, Ministry of Education, Pharmacy School, Shihezi University, Shihezi 832003, China; 5 Research Division of Medicinal Plants, Research Institute of Forests and Rangelands, Agricultural Research Education and Extension Organization (AREEO), Tehran P.O. Box 13185-116, Iran; sefidkon@rifr-ac.ir

**Keywords:** *Ferula*, pharmacology, chemical composition, Apiaceae, Q-marker

## Abstract

The genus of *Ferula* belongs to the family Apiaceae, and many *Ferula* plants are used as traditional Chinese medicines. *Ferula* plants were initially identified as early as the “Newly Revised Materia Medica” written in the Tang Dynasty (AD 659), and several of them are also recognized as the traditional medicines of the Uygur, Kazakh, and Mongolian. *Ferula* plants are distributed in China, Russia, India, Africa, Central Asia, and other places. Currently, the chemical components derived from *Ferula* plants are mainly coumarins, sesquiterpenes, and volatile oils. *Ferula* plants can exhibit diverse pharmacological activities such as anti-allergy, analgesia, relieving cough, anticoagulation, and anti-tumor. Therefore, this article summarized the domestic research conducted on the genus *Ferula*, appropriately combines the research status of the foreign genus *Ferula*, and describes the chemical composition, biological activity, toxicity issues, and Q-marker prediction. In addition, all the related studies about the genus *Ferula* are summarized by analyzing the various databases such as CNKI, Wanfang data, PubChem and SciFinder.

## 1. Introduction

**Figure 1:**
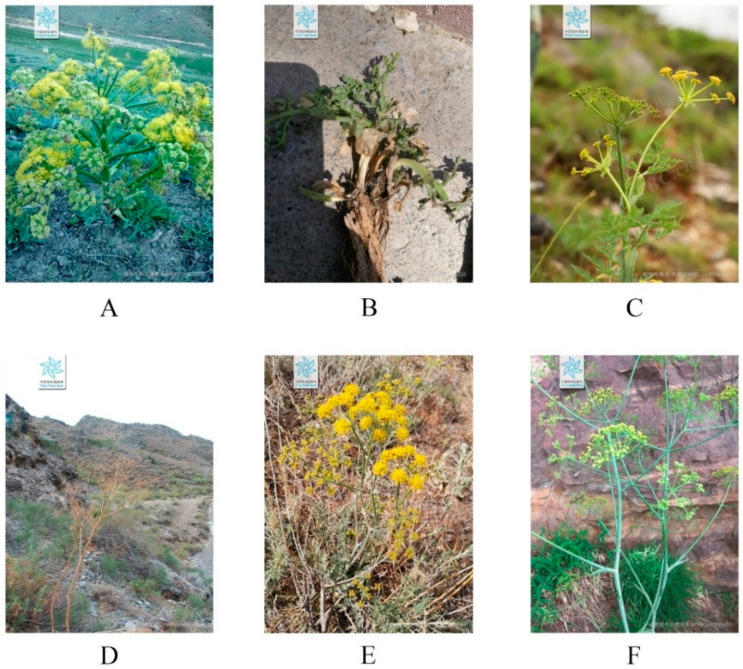
Field morphological pictures of several *Ferula* plants. (**A**). *Ferula sinkiangensis* [5]; (**B**). *F. fukanensis* [6]; (**C**). *F. kingdon-wardii* [7]; (**D**). *F. songarica* [8]; (**E**) *F. syreitschikowii* [9]; (**F**). *F. licentiana* [10]. ((**A**–**F**) are from the Plant Photo Bank of China, http://www.iplant.cn/, 16 June 2023).

*Ferula* is a tall perennial herb which belongs to the Apiaceae family, and these plants are commonly characterized by a peculiar pungent garlic odor. There are more than 180 species of *Ferula* found worldwide [1] and they are mainly located in Central Asia, the Middle East, Siberia, and South Asia [2]. In China, *Ferula* is mainly distributed in Xinjiang, and a small number of *Ferula* plants are also found in Gansu, Shanxi, Inner Mongolia, and Tibet [3,4]. The genus Ferula in China primarily comprises more than twenty species, with a few representative plants shown in Figure 1.

The plants belonging to the genus *Ferula* (Apiaceae) have been implicated in the treatment of various ailments and have a long history of use in many traditional medicines. For instance, Nepali people use *Ferula* in their daily diets [11,12], and it is used locally as a traditional remedy in Saudi Arabia for the treatment of skin infections [13]. In addition, *Ferula* plants are widely used in India in food and as a medicine in Indian systems of medicine such as Ayurveda [14]. Interestingly, in China, *Ferula* plants are also used as an Uyghur medicinal herb, and have a long history of beneficial application [15]. Ferula has already been added to the Uyghur Medicine Criteria [16] and the calendar version of the Chinese Pharmacopoeia [17]. In other ethnic groups and regions of China, *Ferula* is often used as traditional medicines. The traditional uses of *Ferula* include beneficial effects against stomach pain, flatulence, intestinal parasites, indigestion, asthma, and flu [18]. For instance, the Kazakhs have used *F. soongarica* to treat headaches, colds and stomach aches, *F. caspica* can treat nervous breakdown, and *F. lehmannii* is used as an anti-parasitic, for anti-malnutrition and to cure cold and pain in the heart and abdomen [12,15]. A number of modern pharmacological and biological studies have indicated that *Ferula* has anticancer [19,20], anticarcinogenic [21], antimicrobial [22], antidiabetic [23], anti-flu [24], anti-inflammatory [25,26], and cardiovascular system effects [27], as well as use against Alzheimer’s disease [28,29] and in anti-neuropathic pain [30] activities.

Ferula contains a variety of chemical compounds, primarily coumarins [31], particularly sesquiterpenes coumarins [32], volatile oils [33], sulfur-containing compounds [34], and aromatic compounds [35], all of which exhibit diverse biological activities. However, the Q-marker prediction studies of *Ferula* are relatively scarce. *Ferula* plants of China are mainly distributed in Xinjiang, and it is less commonly present in other regions. Thus, in addition to the over-exploitation and collection of *Ferula* resources, the quality of Ferula herbs available on the market is a matter of concern due to the prevalence of counterfeits and inferior species that are accessible to consumers. Furthermore, the genus *Ferula* seed plant breeding has been continuously shrinking due to the rapid land development, irrigation, highway building, leading to significant decrease in *Ferula* resources. Therefore, novel strategies have to be developed to protect the resources of *Ferula* plants.

Traditional Chinese medicines (TCMs) play an important role in China’s modern health care system and have been found to be efficacious in clinical practice. However, due to the lack of criteria for the selection of quality control indicators, both the certification and efficacy of herbal medicines remain unclear [36,37]. Therefore, new approaches are needed to establish Q-markers for determining the quality standards of traditional Chinese medicines, and for identifying the appropriate chemicals present in plants to use them as potential quality indicators [38,39].

There are quite a few Q-marker studies reported for the genus *Ferula*. Thus, the identification of the various markers for the genus *Ferula* by the Q-marker method is essential for the identification of its authenticity and the conservation of the plant resources.

## 2. Distribution of *Ferula* Plants in China

There are more than 180 species of genus *Ferula* in the worldwide, and more than 20 species have been recorded in China. The genus *Ferula* resources are mainly distributed in Xinjiang and a small number of other provinces in China (Table 1). Most *Ferula* plants are distributed at altitudes between 400 and 3500 m, and the distribution environment is mainly the desert and mountainous areas. However, in recent years, due to the destruction of the plant resources of the genus *Ferula*, wild *Ferula* is nearly extinct.

## 3. Research on the Chemical Composition of *Ferula* Plants in China

### 3.1. Coumarins

**Figure 2:**
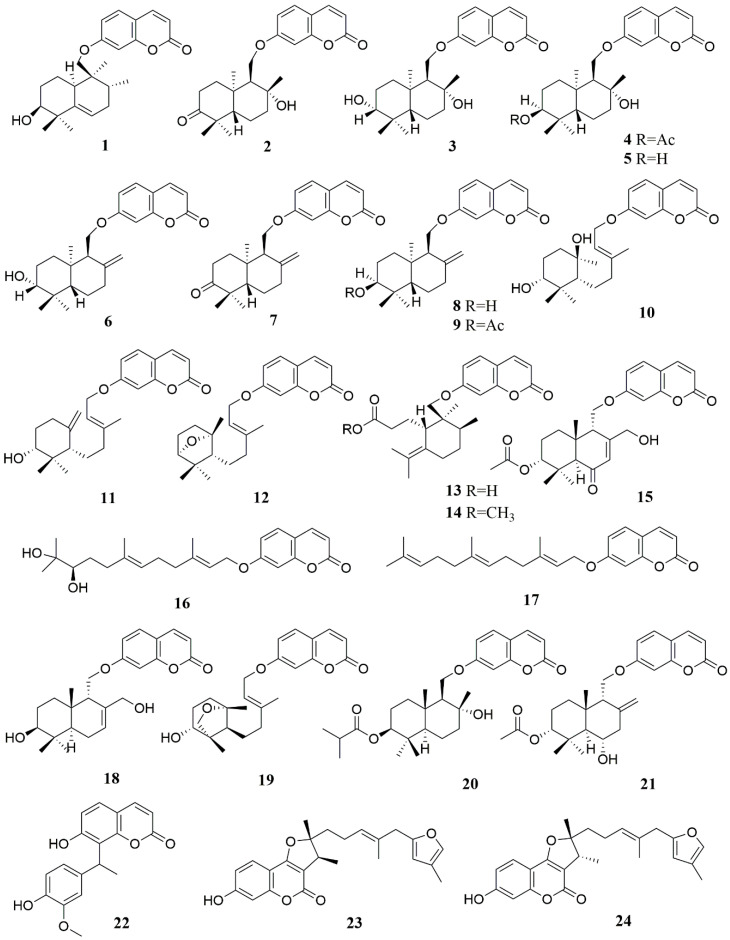
Chemical structures of compounds **1**–**24** from *Ferula* plants.

**Figure 3:**
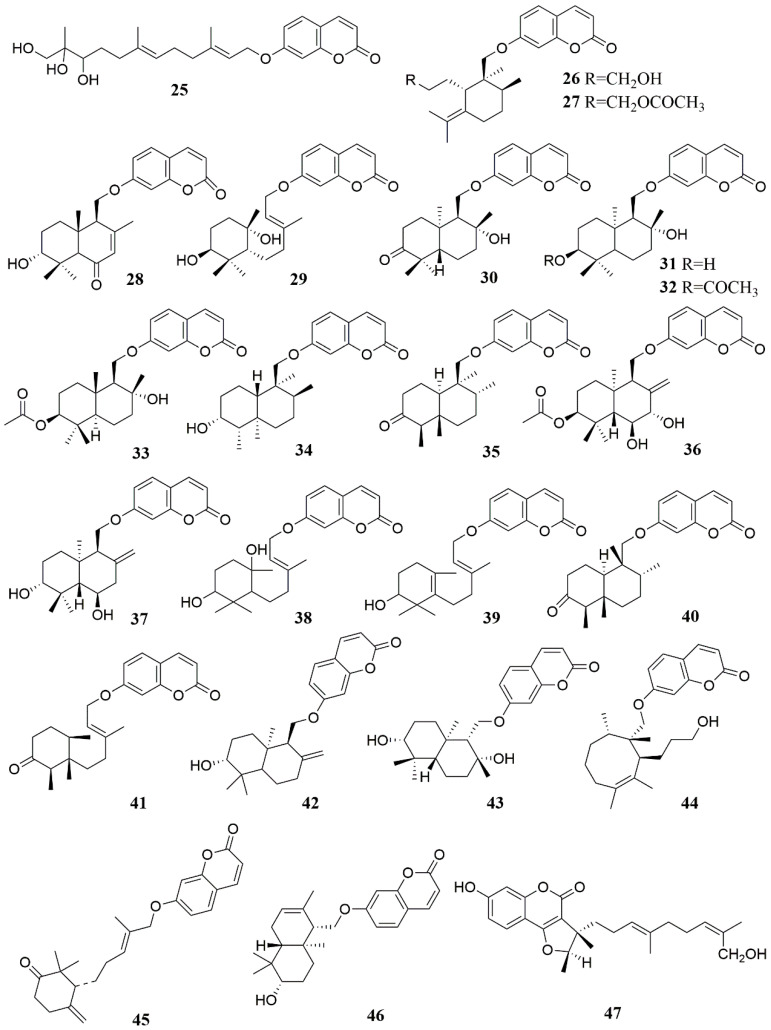
Chemical structures of compounds **25**–**47** from *Ferula* plants.

**Figure 4:**
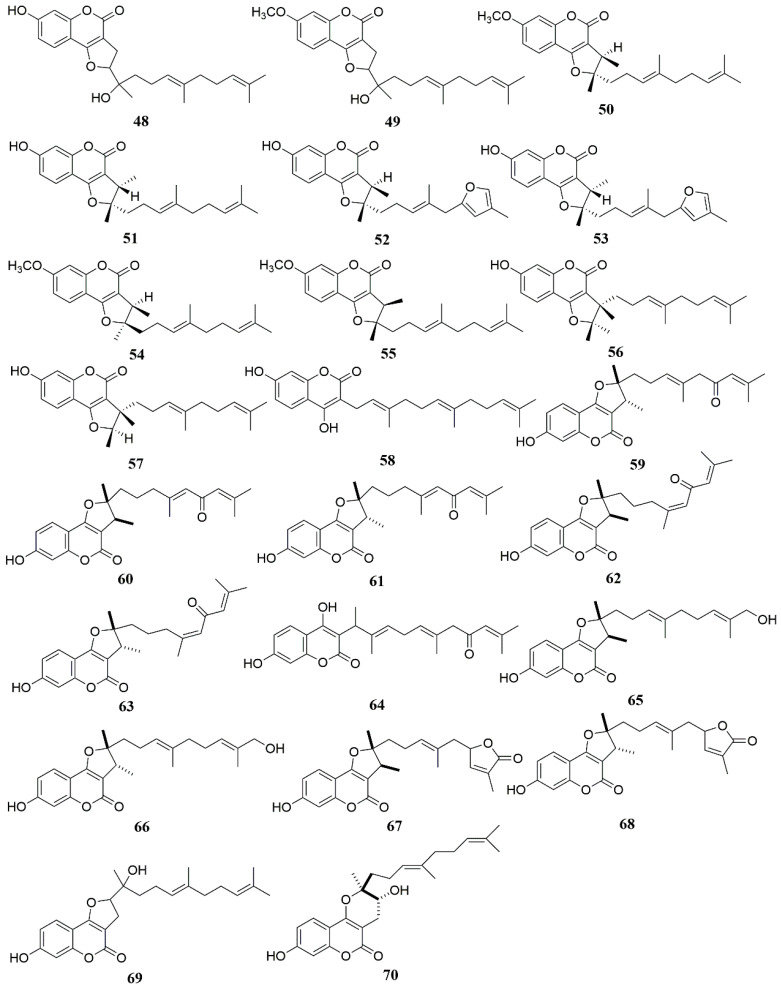
Chemical structures of compounds **48**–**70** from *Ferula* plants.

The full name of 7-hydroxycoumarin is 7-hydroxycoumarin O(7)-glucosiduronic acid. It is a beta-D-glucosiduronic acid. Coumarins are found in the genus *Ferula*, and most of them are derivatives with 7-hydroxycoumarin as the parent nucleus [56]. Coumarins in the genus *Ferula* can be further divided into different types of coumarins according to their substituents, mainly including sesquiterpene coumarins and monoterpene coumarins. Among them, sesquiterpene coumarins can be divided into bicyclic sesquiterpene coumarins (A), monocyclic sesquiterpene coumarins (B), and straight-chain sesquiterpene coumarins (C), in addition to some furan coumarins (D) [57] and other coumarins (E) (Table 2, Figure 2, Figure 3 and Figure 4). Researchers obtained a large number of coumarin-like substances from *Ferula sinkiangensis*, *F. lehmannii*, *F. feruloides* and *F. fukanensis* [58,59,60,61,62,63,64,65,66].

### 3.2. Sesquiterpenoids and Their Derivatives

**Figure 5:**
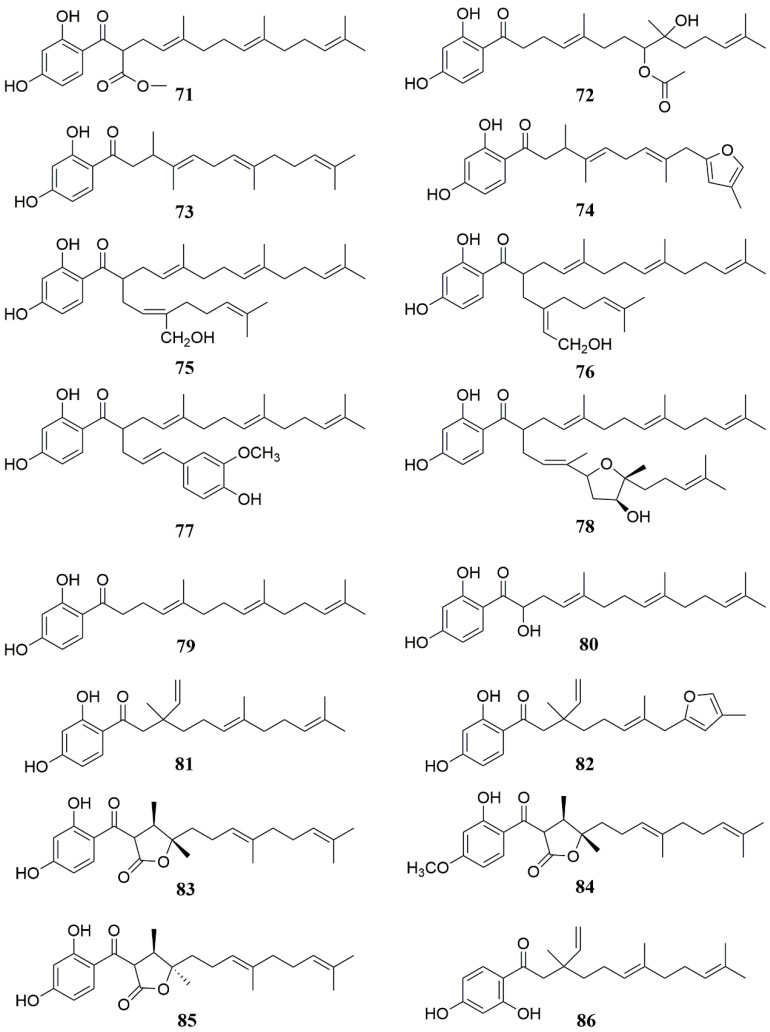
Chemical structures of compounds **71**–**86** from *Ferula* plants.

**Figure 6:**
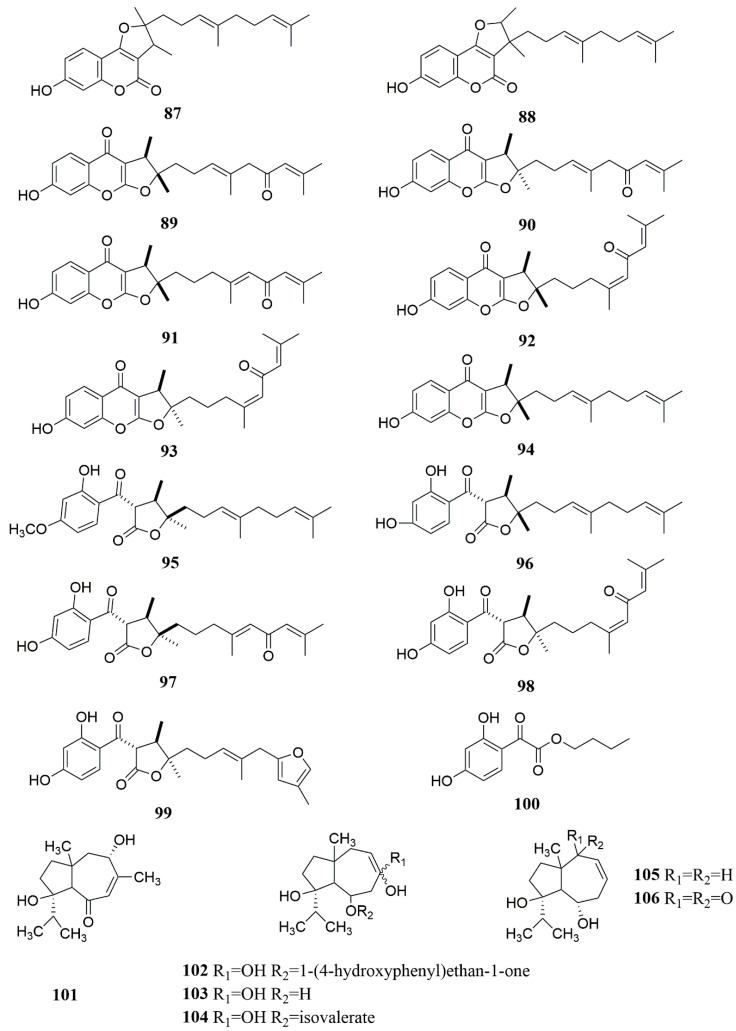
Chemical structures of compounds **87**–**106** from *Ferula* plants.

In addition to the above-mentioned coumarins, sesquiterpenes are the second most abundant substances in *Ferula* plants. The sesquiterpenes of the genus *Ferula* are mainly carotene-type sesquiterpene parent nuclei, but carotene-type sesquiterpenes are rare in domestic *Ferula* plants and are mostly concentrated in foreign *Ferula* plants. The researchers extracted the following sesquiterpenoids from *F. ferulaeoides, F. caspica, F. fukanensis* and *F. jaeschkeana* [67,68,69,70,71] (Table 3, Figure 5 and Figure 6).

### 3.3. Volatile Oil

**Figure 7:**
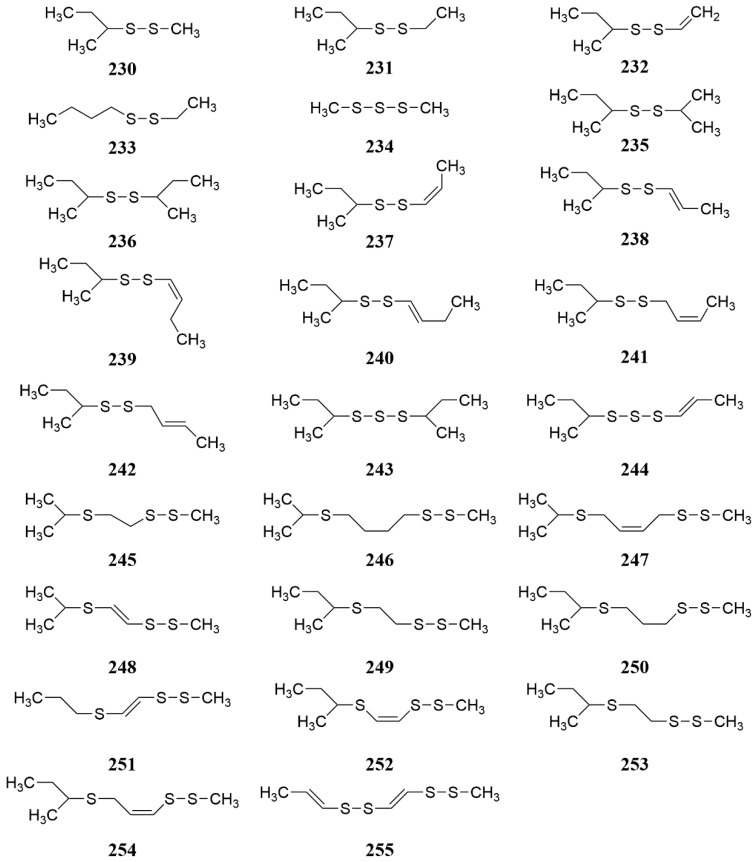
Chemical structures of compounds **230**–**255** from *Ferula* plants.

The volatile oils in genus *Ferula* are mainly terpenoids and polysulfide compounds [72]. Among the genus *Ferula* of China, the number of terpene components can reach more than 80% in volatile oils, mostly are monoterpenes and sesquiterpenes, while polysulfide compounds are dominated by disulfides, trisulfides, bis-disulfides, and thio-disulfides. A large amount of volatile oil was extracted from *F. sinkiangensis, F. fukanensis, F. ferulaeoides, and F. ovina* [73,74,75,76] (Table 4). Min Zhi-da et al. [77] identified 26 polysulfides by gas chromatography-mas spectrometry (GC-MS (CI/EI)) in *F. sinkiangensis* and *F*. *fukanensi* (Figure 7).

### 3.4. Aromatic Compounds

**Figure 8:**
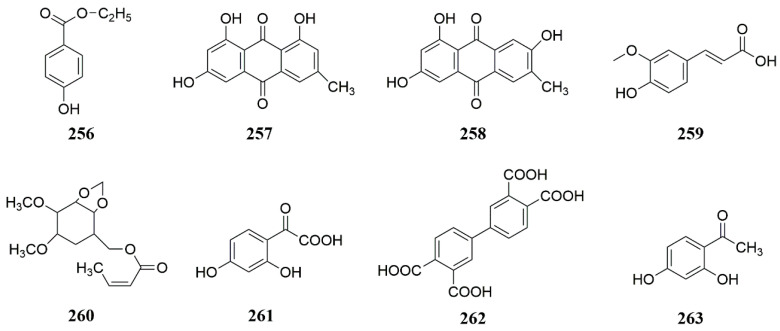
Chemical structures of compounds **256**–**263** from *Ferula* plants.

Studies showed that ethyl p-hydroxybenzoate (**256**), emodin (**257**), and 1,3,7-trihydroxy-6-methyl-xanthone (**258**) were isolated from *F. fukanensis* [78]. Ferulic acid (**259**) was isolated for the first time from *F. sinkiangensis* [62]. 4,5-Dimethoxy-2,3-methylenedioxyphenylpropane-7-adamate was isolated from *F. licentiana* [79] and named as Tong Shan ferulic acid A (**260**). 2,4-Dihydroxy-α-oxo-phenylacetic acid (**261**), 3,3′,4,4′-biphenyltetracarboxylic acid (**262**) and 2,4-dihydroxybenzophenone (**263**) were isolated from the rhizome of *F*. *songarica* [80]. 2,4-Dihydroxy-α-oxo-phenylacetic acid was first isolated from *F. songarica* and 3,3′,4,4′-biphenyltetracarboxylic acid was a newly discovered compound (Figure 8).

### 3.5. Other Substances

**Figure 9:**
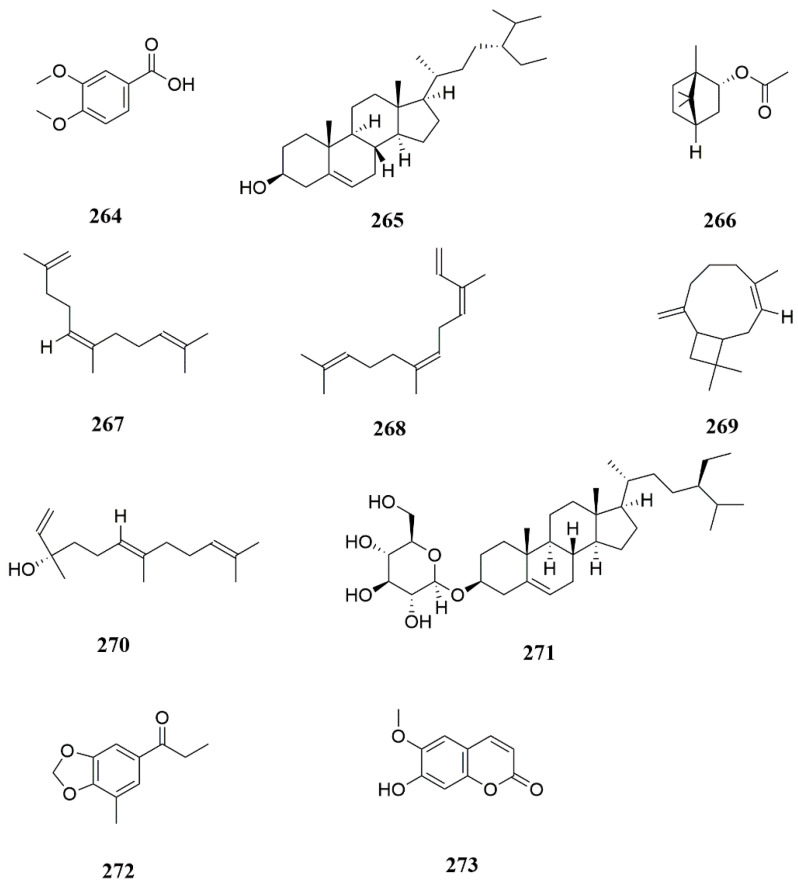
Chemical structures of compounds **264**–**273**.

Veratric acid (**264**) and β-sitosterol (**265**) were isolated from *F. licentiana* [81]. Acetophenone compounds such as (1*S*,2*R*,4*S*)-(−)-borneol acetate (**266**), (5*Z*)-2,6,10-trimethyl-1,5,9-undecatriene (**267**), (*Z*)-β-farnesene (**268**), humulen-(v1) (**269**), ±-trans-nerolidol (**270**), and carotene (**271**) were isolated from *F. ferulaeoide* [82,83] (Figure 9). Seventeen amino acids, including lysine, histidine, arginine, aspartic acid and threonine, were obtained from the roots and leaves of *F. lehmannii* Boiss [84]. Studies have shown that a series of trace elements including potassium, aluminum, calcium, copper, magnesium, barium, cadmium, and cobalt were isolated from *F. sinkiangensis* [85]. Radiatinol (**272**) and scopoletin (**273**) were isolated from *F. dissecta* [86].

## 4. Advances in the Bioactivity of *Ferula* Plants in China

With the gradual increase in domestic research in the field of biological activity of the genus *Ferula*, the bioactive compounds were isolated from the Ferula plant by various applied extraction techniques [87], and a number of different compounds with anti-tumor [88], anti-allergy, anticoagulation [89], bacterial inhibition [90], action in the gastrointestinal system [91], nervous system [92], cardiovascular system [93,94], and other pharmacological activities have been found in the genus *Ferula* [95,96,97].

### 4.1. Anticancer Activity

The main chemical components of *Ferula* plants that exert anti-tumor effects are ferulic acids [98], sesquiterpenoids [99], and volatile oils [100]. For instance, Wu Jing [101] found that the inhibition rate of A549 lung cancer xenografts in nude mice gradually increased with increase in dose of ferulic acid administration. The low (25 mg/L), medium (50 mg/L) and high (100 mg/L) dose groups inhibited the expression levels of both mTOR protein and mRNA in the tumor tissues of the nude mice, and decreased the expression levels of Ki-67 protein, which decreased with increasing dose, and also enhanced the expression levels of Caspase-3 protein. The investigators suggested that ferulic acid can attenuate growth of lung cancer probably by downregulating mTOR protein expression, inhibiting Ki-67 expression and promoting Caspase-3 levels. The petroleum ether, n-butanol, ethyl acetate, trichloromethane, and aqueous fractions, as well as volatile oil and 95% ethanol extract of *F. ferulaeoides* were screened for their pharmacological activities, among which trichloromethane and ethyl acetate fractions exhibited certain inhibitory effects on five distinct gastric cancer cell lines, whereas trichloromethane fractions showed the strongest inhibitory effect on gastric cancer cells SGC-7901 (IC_50_: 7.98 ± 2.62 mg/L). In addition, volatile oil displayed strong inhibition of cell proliferation on gastric cancer cells AGS (IC_50_: 8.73 ± 0.55 mg/L), but n-butanol fraction and aqueous fraction showed no significant inhibitory effects on these five gastric cancer cell lines (AGS,MKN-45,BGC-823,MGC-803,SGC-7901) [102]. A sesquiterpene coumarin-like substance,2,3-dihydro-7-hydroxy-2R*,3R*-dim-ethyl-2-[4,8-dimethyl-3(*E*),7-nonadienyl]-furo[3,2-*c*]coumarin (DAW22), was isolated from *F. ferulaeoides* root, and was subsequently reported to induce apoptosis in C6 glioma cells through the mitochondria-mediated, endoplasmic reticulum stress and death receptor pathway. The dosing concentration is 20 µM [103]. It also suppressed the proliferation of five different established human Malignant peripheral nerve sheath tumors (MPNST) cancer cell lines (STS-26T, S462, S462-TY, ST8814, T265) by promoting their apoptosis [104]. Moreover, experimental animal studies have revealed that in an immunocompromised nude mouse model with subcutaneous transplantation of STS-26T cells, when DAW22 was administered intraperitoneally at a daily dose of 60 mg/kg/day for 4 weeks, it significantly inhibited tumor xenografts compared to the control group. It was found by Western blotting analysis, that DAW22 inhibited phosphorylation of AKT and ERK and reduced the level of Non-phospho (Active) CTNNB1, there by resulting in an inhibitory effect on cell proliferation and suppression of tumor growth in for MPNST cell lines. These results supported the use of DAW22 as an alternative therapeutic compound to MPNST which can effectively produce anticancer effects by affecting multiple signaling pathways in tumor disease progression. 8-*p*-Hydroxybenzoyl tovarol (TAW) was isolated from the roots of *F.dissecta*, and found to inhibit the growth of human cervical cancer HeLa cells by promoting protective autophagy, and in the future, advanced molecular dynamics techniques and free energy calculations and reverse molecular docking can be used to reveal the effects of the major coumarins, sesquiterpenoids, and terpenoids in the genus *Ferula* on the anticancer mechanism, and there are already documented studies of other plants using similar measurement techniques [105,106].

### 4.2. Antibacterial Activity

Extraction studies on three distinct kinds of dry roots of *F. sinkiangensis*, *F. lehmannii* Boiss. and *F. ferulaeoides* revealed that the alcoholic and macerated extracts displayed antibacterial effects on *Staphylococcus aureus*, *Bacillus subtilis*, and Sporosarcina. The MIC about *F. sinkiangensis* root’s alcoholic extracts for three bacteria were 31.25 mg/mL, 3.92 mg/mL, and 1000 mg/mL. The MIC about *F. sinkiangensis* root’s macerated extracts for three bacteria were 1000 mg/mL, 1000 mg/mL, and 1000 mg/mL. The MIC about *F. ferulaeoides* root’s alcoholic extracts for three bacteria were 3.91 mg/mL, 3.91 mg/mL, and 15.63 mg/mL. The MIC about *F. ferulaeoides* root’s macerated extracts for three bacteria were 500 mg/mL, 500 mg/mL, and 1000 mg/mL. The MIC about *F. ferulaeoides* root’s alcoholic extracts for three bacteria were 7.81 mg/mL, 3.91 mg/mL, and 15.63 mg/mL [107]. A total of 27 compounds were extracted and isolated from *F. ferulaeoides*, and 7 of the 27compounds were found in the plant for the first time. These 27 compounds were screened for their potential antibacterial activity, and only a few coumarins among the 27 compounds were found to affect multi-drug resistant (MDR) and methicillin-resistant Staphylococcusaureus (MRSA) bacteria, and six of them showed inhibition against tetracycline-resistant strain XU212 [108]. In addition, three new sesquiterpene derivatives were obtained from *F. ferulaeoides* [109] and it was observed that their minimum inhibitory concentrations (MIC) ranged from 0.5–128 μg/mL. It was found that the volatile oil of the seeds of *F. olivacea* exhibited an inhibitory effect on the Gram-positive bacteria such as *Enterococcus faecalis* and *S. aureus* [110,111].

### 4.3. Anti-Allergic, Anti-Inflammatory, and Immunosuppressive Effects

Using sodium cromoglycate (10 mg/kg) as the control group and volatile oil of *F. sinkiangensis* (50 mg/kg) as the experimental group, experiments were conducted to evaluate the potential effect of passive skin allergic reaction in mice, the effect of Arthus reaction in rabbits, and the antigen sensitization confrontation in the sensitized guinea pigs [112]. It was found that the *F. sinkiangensis* volatile oil (200 mg/kg) could significantly inhibit Ig E response to allergic reactions, and the degree of effect was similar to that of sodium cromoglycate. Ferulic acid can also reverse skin sensitization in rats and suppress the delayed hypersensitivity reactions in mice, thus indicating that ferulic acid has inhibitory effect on allergic reactions [113]. In another study, designed to examine the impact of the raw juices of *F. sinkiangensis*, *F. conocaula,* and *F. feruloides*, it was found that all three types of *Ferula* plants exhibited significant inhibitory effects on foot topography swelling (the dosing concentrations were *F. sinkiangensis*: 90 mg/kg, *F. conocaula*: 90 mg/kg, *F. feruloides*: 80 mg/kg)and capillary permeability (the dosing concentrations were *F. sinkiangensis*: 34 mg/kg, *F. conocaula*: 34 mg/kg, *F. feruloides*: 30 mg/kg) in rabbits induced by p-hornwort, and on the delayed hypersensitivity (the dosing concentrations were *F. sinkiangensis*: 98 mg/kg, *F. conocaula*: 97 mg/kg, *F. feruloides*: 78 mg/kg) and serum hemolysin formation (the dosing concentrations were *F. sinkiangensis*: 98 mg/kg, *F. conocaula*: 97 mg/kg, *F. feruloides*: 78 mg/kg) in mice induced by sheep erythrocytes or dinitrochlorobenzene [114].

### 4.4. Anticoagulant Effect

The extract of *F. lehmannii* was obtained by using 95% ethanol, and an anticoagulant assay was then performed. The experimental results revealed that *F. lehmannii* extract inhibited the endogenous coagulation pathway and showed an inhibitory effect on the activity of thrombinogen. The dosing concentrations were: the high-dose group (100 mg/kg), the medium-dose group (50 mg/kg), and the low-dose group (25 mf/kg) [115]. Radiatinol and scopoletin were isolated from *F. dissecta*, and both these compounds acted in the endogenous coagulation pathway and prolonged the prothrombin time as well as activated partial thromboplastin time at different concentrations [86].

### 4.5. Effects on the Cardiovascular System

Interestingly, another study showed that after ferulic acid was administered to C57 mice in the heart failure preclinical model, the mice in the heart failure administration group exhibited different degrees of improvement in cardiac left ventricular ejection fraction, end-systolic and end-diastolic internal diameter changes, collagen area percentage changes, and protein expression levels compared to the control mice. Ferulic acid could improve ventricular remodeling to some extent [116]. In addition, studies conducted with the hydroalcoholic extracted fractions and the aqueous decoction of *F. sinkiangensis* revealed that they could reduce the heart amplitude and enhance the heart rate in isolated frog hearts [117].

### 4.6. Effects on the Gastrointestinal Tract

It has been reported in the literature that *F. sinkiangensis* has been used to treat stomach disorders in Xinjiang [118]. The volatile oil and resin of *F. sinkiangensis* were found to be effective in three distinct gastric ulcer models, among which the volatile oil was found to be relatively better [119]. The rat model of acetic acid-injected gastric ulcer was established, and the model was tested by administering *F. sinkiangensis* original herbs and *F. sinkiangensis* prepared herbs by a folk concoction (0.048 g/mL), frying method (0.048 g/mL), a vinegar moxibustion method (0.048 g/mL), and a boiling method (0.048 g/mL). It was found that the pH value of the rat stomach increased significantly, but the area of gastric ulcer decreased markedly after the administration of *F. sinkiangensis* original herbs and *F. sinkiangensis* prepared herbs [120]. In addition, studies on the raw juices of *F. feruloides* and *F. sinkiangensis* revealed that these juices can display inhibitory effects on gastric ulcer models in rats with acetylsalicylic acid and can effectively suppress the autonomous activity of smooth muscle in isolated intestinal tubes of rabbits [121]. The compounds were isolated from the *F. sinkiangensis* resin and they were analyzed in anti-ulcer vivo activity experiments. It was observed that compared with the positive control group famotidine, CHCl3 extract (1.3515 g/kg) showed the best anti-ulcer activity [122].

### 4.7. Action on the Nervous System

A network pharmacological analysis of *Ferula* resulted in identification of 12 key active components against Alzheimer’s disease, including Farnesiferol A, Farnesiferol B, conferol, and ferulic acid. It was predicted that *Ferula* can predominantly act through by modulating 14 key targets in the cholinergic synaptic signaling pathway and AD signaling pathway [123]. A bioactivity-oriented study on *F. sinkiangensis* showed that *F. sinkiangensis* significantly inhibited NO production induced by over-activated microglia and exhibited anti-neuroinflammatory effects [58]. Interestingly, a compound, Kellerin, extracted from *F. sinkiangensis*, was found to produce substantial effects in the rat middle cerebral artery occlusion (MCAO) model, lipopolysaccharide (LPS)-activated microglia model, mouse bilateral common carotid artery occlusion (BCCAO) model and lipopolysaccharide (LPS)-activated microglia model. Kellerin was found to markedly improve the neurological outcomes, reduce the size of cerebral infarcts and decrease brain edema in the rat MCAO model, and the dosing concentrations were: the low-dose group (3.5 mg/kg), the medium-dose group (7.0 mg/kg) and the high-dose group (14.0 mg/kg). Moreover, in the pathological conditions of focal cerebral ischemia, Kellerin could attenuate neuronal damage as well as microglial activation. In addition, in vitro study which LPS-stimulated BV2 cells, revealed that Kellerin protected the neuronal cells from injury by inhibiting microglia activation. Kellerin also reduced levels of pro-inflammatory cytokines, inhibited the NF-κB signaling pathway, and decreased ROS production as well as NADPH oxidase activity, and the [124]. Kellerin was found to alleviate the cognitive impairment, reduce neuronal loss, inhibit microglia activation, and convert microglia from a pro-inflammatory M1 phenotype to an anti-inflammatory M2 phenotype in BCCAO mice. In addition, in another in vitro study, Kellerin was found to modulate the microglia polarization and inhibit NLRP3 and MAPK signaling pathways after LPS treatment [125].

### 4.8. Other Pharmacological Effects

Furthermore, studies have shown that *Ferula* resin can exhibit anti-pregnancy, anti-implantation and pregnancy termination effects [126,127]. It can also affect estrogen and progesterone levels in infertile mice and rabbits, thus modulating the smooth muscles of the uterus in mice and rabbits [128]. In another study, d-galactosamine/lipopolysaccharide acute liver injury model was established, and by comparing the d-galactosamine/lipopolysaccharide model group, the biphenyldiglyceride group and the high-, medium- and low-dose ferulic acid groups, it was observed that all the three ferulic acid groups could significantly decrease the activity of enzymes AST and ALT in mouse hepatocytes, increase the activity of antioxidant enzyme SOD as well as peroxidase GSH-Px in mice, and decrease the MDA content, thereby suggesting that ferulic acid exhibited a protective effect on acute liver injury [129]. The petroleum ether, ethyl acetate, n-butanol and methanol fractions of *F. sinkiangensis* displayed no substantial effect on the lipase activity, whereas the ethyl acetate, n-butanol and methanol fractions lowered total cholesterol in HepG-2 cells and triglycerides in non-alcoholic fatty liver model cells. Thus, it was hypothesized that *F. sinkiangensis* has certain lipid-regulating effects [130]. Four coumarin-like compounds were extracted from *F. moschata* showed anti-HIV activity [131].

### 4.9. Toxicological Studies

Acute toxicity experiments were conducted on the volatile oils of *F. feruloides* and *F. teterrima*, and the findings revealed that the LD50 of the volatile oil of *F. feruloides* was 10.24 g/kg and the LD50 of the volatile oil of *F. teterrima* was 491.61 mg/kg. After analyzing the volatile oils of both, it was found that the volatile oil of *F. teterrima* contained more polysulfides, accounting for 60% of the volatile oil, but no polysulfides were detected in the volatile oil of *F. feruloides*. Polysulfide can cause irritation and possess a garlic-like odor, so it was presumed that polysulfide was more likely to be a toxic component in *F. teterrima* [132]. However, the acute toxicity studies with polysulfide has not been reported previously in the literature. It was found that goats fed with *F. fukanensis* (2.5 g/kg) for 15 days showed obvious signs of toxicity, such as loss of appetite, anorexia, diarrhea, vitamin K-dependent reduction in coagulation factors, the toxic component of which may be possibly coumarins, but no obvious symptoms were found in these animals, such as impaired liver and platelet function [133]. Acute toxicity tests were conducted on *F. sinkiangensis* and *F. fukanensis* [134], and the LD50 values were calculated by Kou’s method. The LD50 values of *F. sinkiangensis* volatile oil aqueous suspension and *F. fukanensi* volatile oil aqueous suspension were found to be 2.82 g/kg and 1.55 g/kg; and the LD50 values of *F. sinkiangensis* volatile oil emulsion and *F. fukanensi* volatile oil emulsion were separately 0.39 g/kg and 0.41 g/kg^−1^. After analyzing ten batches of *F. sinkiangensis* test samples [135], two of them were observed to contain different ferulic acid contents. No. 1 *F. sinkiangensis* with (0.775 ± 1.44) mg/g and No. 5 *F. sinkiangensis* with (0.279 ± 1.63) mg/g, were selected and subjected to the toxicity studies by SRB method. The proliferation rates of No. 1 *F. sinkiangensis* and No. 5 *F. sinkiangensis* on the rat renal NRK cells was found to gradually increase with increasing drug concentrations, with IC50 values of No. 1 *F. sinkiangensis* at 60.60 mg/Land No. 5 *F. sinkiangensis* at 39.90 mg/L. The comparison of the IC50 values showed that the cytotoxicity of No. 1 *F. sinkiangensis* was significnatly less than that of No. 5 *F. sinkiangensis*, but the ferulic acid content of No. 1 *F. sinkiangensis* was significantly higher than that of No. 5 *F. sinkiangensis*, thus indicating that the toxicity of *F. sinkiangensis* may be possibly related to its chemical composition.

## 5. Quality Marker (Q-Marker) Prediction Analysis

With the depletion of the domestic *Ferula* plant resources, the phenomenon of substandard and uneven quality of *Ferula* plants in the market needs to be urgently solved. Thus, the quality markers (Q-marker) [136] need be established for the effective protection of the domestic *Ferula* plant resources. At present, the Chinese Pharmacopoeia 2020 edition stipulates that the sources of medicinal *Ferula* plant is the resin of *F. sinkiangensis* and *F. fukanensi* and requires that their quality marker is ferulic acid. However, ferulic acid is widely present in different plants such as *Angelica sinensis* [137], *Ligusticum chuanxiong* [138], *Cimicifugafoetida L*. [139], *Ligusticum* [140] and *Fructus Toosendan* [141]. It is also an important active component of many traditional Chinese medicines [142]. A large amount of sesquiterpene coumarins are found in *Ferula* plants, and they may be used as unique chemical components which can effectively help to identify *Ferula* plants by the Q-marker method.

### 5.1. Q-Marker Prediction Analysis by Kinship and Chemical Composition Specificity of Ferula Plants

The global distribution of *Ferula* plants is widespread in Central Asia, Iran and Pakistan. It has been reported that during the course of foreign research on the chemical composition of *Ferula* plants, it was observed that genus *Ferula* contained a large number of sesquiterpene coumarins with umbelliferous lactones as the parent nucleus [143], and Ferulenol was the most abundant and the first isolated coumarin from *Ferula* [144]. Although various sesquiterpenes and coumarins are widely distributed in the Apiaceae family, they constitute the characteristic chemical constituents of genus *Ferula*, and can be used as important evidence of the affinities of *Ferula* plants. They could also form a sound basis for the chemical taxonomy of genus *Ferula* [145], such as Farnesiferol A, Farnesiferol B, Farnesiferol C, and DAW22, because sesquiterpene coumarins are widespread in different plants of the genus *Ferula*. Additionally, the pharmacopoeia mentions ferulic acid as potentially another characteristic chemical constituent of the genus *Ferula*.

### 5.2. Q-Marker Prediction Analysis by Chemical Composition Validity

As an emerging method to evaluate and control the quality of Chinese herbal medicine Q-markers is essential to predict both the effectiveness and safety of Chinese herbal medicines. Therefore, Q-marker analysis of Chinese herbal medicines should be combined with the effectiveness of the target herbal medicines to facilitate better research on the effectiveness of Chinese herbal medicines [146,147]. Because of the presence of the different coumarin sesquiterpenoids in large quantities in the genus *Ferula*, most studies on the chemical composition effectiveness and safety of *Ferula* resources have primarily focused on these compounds, which are expected to become quality markers of *Ferula* plants.

#### 5.2.1. Q-Marker Prediction Analysis by Traditional Drug Properties

The medicinal properties of *Ferula* plants have been recorded as Ku (bitter), Xing (pungent) and Wen (warm), as well as Guipi and Weijing (benefit for the spleen and stomach) in the Chinese Pharmacopoeia Edition (2020). The chemical compositions of the various bitter medicines in Chinese medicine are mainly alkaloids, glycosides, terpenoids and other bitter substances [148,149]. Terpenes have a bitter taste primarily due to their chemical structure with chelating structures such as lactones, endo-acetals, endo-hydrogen bonds and glycosidic groups [150]. The sesquiterpene coumarins, which are found in relatively large quantities in the genus *Ferula*, are based on 7-hydroxyumbelliferolactone as the parent nucleus. A number of prior studies have shown that terpenes and volatile oils are found in ample quantities in herbs with the Xing (pungent) taste [151], whereas coumarin sesquiterpenes are similarly abundant in the genus *Ferula*. In addition, the main chemical components of genus *Ferula*, such as Farnesiferol A, Farnesiferol B, Farnesiferol C and DAW22, are all coumarin sesquiterpenoids.

#### 5.2.2. Predictive Analysis by the Q-Marker of Traditional Efficacy

The effects of *Ferula* plants have been recorded in the Chinese Pharmacopoeia 2020 edition and found to be useful as digesting aid, relief of symptoms, dispersal of agglomerates as well as insecticide. They are also used for the treatment of excessive meat consumption, leading to stagnation and masses in the abdomen, and treating bruises, intestinal parasites and the abdominal pain [17]. Their effects are reported in the Xinxiubencao as an insecticide, deodorization, in elimination of intra-abdominal masses, relief of blood stasis, septicemia and pus, and alleviation of symptoms of poisoning [152]. *Ferula* plants have been also used to treat tuberculosis, eliminate lumps that have accumulated in the abdomen, cure cold, and treat malaria, which are effective against acute gastroenteritis as well as other intestinal diseases. They have a pain-relieving effect on the chest and stomach in addition to pain [153] and similar records have been found in other ethnomedicinal books [154]. Moreover, during the course of modern pharmacological research, the pharmacological effects of *Ferula* plants are similar to those recorded in ancient books, such as anti-allergy, action on the gastrointestinal tract, anti-bacterial as well as antiseptic effects, anticoagulant effects, and effects related to the cardiovascular system. The CHCl3 extracted fraction of *F. sinkiangensis* resin can exhibit significant anti-gastric ulcer activity, and the sesquiterpene coumarins contained in this fraction were mainly Farnesiferol B and Farnesiferol C. In addition, prior studies have shown that the raw juices of *F. sinkiangensis* and *F. feruloides* have certain effects on gastrointestinal smooth muscle inhibition and anti-experimental gastric ulcer, whereas the chemical compositions of these three types mainly consist of the sesquiterpenes, Farnesiferol A [58], Farnesiferol B, Farnesiferol C. Interestingly, in vivo studies have shown that Farnesiferol B can protect the kidney from I/R-induced damage by reducing oxidative stress and inflammation. In vitro, Farnesiferol B was reported to improve macrophage migration by activating TGR5 [155]. Furthermore, DAW22 was shown in the literature to inhibit five human MPNST cancer cell lines and C6 glioma cells to varying degrees. Overall, ferulic acid possesses numerous pharmacological activities, mainly related to anticancer, anti-bacterial, anti-inflammatory, antioxidant, anti-thrombotic actions, and can also exhibit hypolipidemic effects [156,157].

### 5.3. Q-Marker Predictive Analysis by Chemical Composition Measurability

The chemical compositions of Chinese medicines are complex, and therefore the chemical measurability of the subject is reported to be an essential element in the Q-marker study [158]. Thus, based on the previous summary study on the specificity and close linkage of the chemical compositions as well as effectiveness of the domestic plants of the genus *Ferula*, the sesquiterpene coumarins and ferulic acid were further identified as the quality markers of *Ferula* plants by measuring their chemical compositions. The contents of ferulic acid were determined by HPLC on the different parts of *F. sinkiangensis* and the roots and leaves of *F. fukanensis*, and the content of ferulic acid was found to be in the following descending order: gum > root > leaf > stem [159,160], and the content of ferulic acid was also determined by HPLC on the anti-ulcer extract of *F. sinkiangensis* [161]. In addition, the determination of Farnesiferol A, Farnesiferol C and ferulic acid in the ethyl acetate fraction of *F. sinkiangensis* was performed by HPLC [162]. The UPLC method was used to estimate the content of DAW22 in *F. feruloides*. It was observed that the methanolic extract of *F. feruloides* contained more DAW22, and DAW22 was most abundant in *F. feruloides* harvested in the first half of May [163].

## 6. Conclusions

Although the genus *Ferula* is widespread and diverse throughout the world, its distribution in China is concentrated mainly in Xinjiang and is less diverse. *Ferula* is native to Central Asia and Middle East, and especially found in eastern Iran and Afghanistan, and is exported to be present worldwide. Nevertheless, *Ferula* is not native to China, and it has been around for a long time but has become a traditional Chinese herb and ethnic medicine. It plays a vital role in the traditional Chinese medical system.

The document analysis of the China’s *Ferula* resources revealed that there are several studies describing the chemical compositions and biological activities of *Ferula* plants. *Ferula* are mainly composed of coumarins, particularly sesquiterpenes coumarins, volatile oils, sulfur-containing compounds, and aromatic compounds. Interestingly, beneficial pharmacological activity of *Ferula* is not only traditionally used, but its various components have been reported to possess anticancer, anti-diabetic, antibacterial, anti-flu, anti-inflammatory and other effects.

The documented benefits of *Ferula* include the elimination of lumps, deworming, antibacterial and other traditional uses. In addition, some traditional uses of *Ferula* are closely related to the modern research and have clinical relevance. For example, modern phytochemical and pharmacological studies have revealed that DAW22 could target the main components in the MPNST tumorigenic pathways: namely, suppress the phosphorylation of AKT and ERK, and reduce the levels of non-phospho (active) CTNNB1. Moreover, other studies have reported that farnesylate A can facilitate the anticancer drugs to exert substantial cytotoxic effects by inhibiting P-glycoprotein. Another study showed that both Farnesiferol B and Farnesiferol C displayed the best inhibitory effects on P-gp pump efflux and they could be considered as lead scaffolds for further structure modifications. Currently, anticancer activity of *F. ferulaeoides* is being investigated by the author and his colleagues.

Because of the availability of a wide variety of TCM, their quality standards are not uniform, thereby resulting in the gradual increase in defective medicines, which can adversely the efficacy of TCM. Faced with this challenge, in 2016, Academician Changxiao Liu proposed the concept of the Q-marker. A Q-marker can better identify the various characteristic compounds present in TCM and can be used sequentially as a criterion to evaluate their quality and to better identify their authenticity, thus providing quality assurance for the development of TCM. Additionally, it was found that there were only a few selected studies related to Q-markers of domestic *Ferula*. Q-marker studies can be of great help to the breeding, conservation and utilization of the domestic *Ferula*. However, it was observed that the few studies on the Q-markers of domestic *Ferula* were not conducive to the research development of domestic *Ferula* spp. in the context of mixed quality.

Moreover, there are only few toxicological studies on the genus *Ferula* in China. Toxicological studies could be of great help in the clinical analysis of the active components in the domestic *Ferula* plants, and hence toxicological studies about the chemical composition of domestic *Ferula* plants should also be strengthened in the future.

Eventually, *Ferula* plants have a long history of use in China, but *Ferula* plant resources have suffered great damage due to human activities in recent years, resulting in acute shortage of plant resources and protective measures are urgently needed. However, regarding the rich background of biological activities of *Ferula*, it appears that there are still a large number of unaccomplished investigations that need to be conducted in the future. Examples include research on the biological characteristics and genetic variation mechanisms of the genus Ferula, for better germplasm conservation and utilization; research on the medicinal value and pharmacological mechanisms of action of the genus Ferula, to explore its application prospects and value in the field of medicine; research on the cultivation and production techniques of Ferula spp. to improve its yield and quality and to meet market demand; analysis and research on the distribution status of the resources and ecological impact of the genus Abies, and the development of conservation strategies to effectively protect and manage these precious plant resources.

## Figures and Tables

**Table 1:** Distribution table of domestic *Ferula* plants.

No	Plant Name	Major Origins	Growing Environment	Literature
**1**	*F. sinkiangensis* K.M. Shen	Yining, Xinjiang	750~1000 m in alpine meadows, stony slope areas	[40]
**2**	*F. fukanensis* K.M. Shen	Fukang, Xinjiang, Southern Gurbantunggut Desert, Xinjiang	The arid inland Gobi Desert	[41]
**3**	*F. ferulaeoides* (Steud.) Korovin	Junggar Basin and Tacheng Basin, Xinjiang	Growing in 430~1000 m sand dunes, sandy land, the environment is mostly desert land	[42]
**4**	*F. conocaula* Korov.	Ucha County, Xinjiang	Elevation 2700~3000 m in mountain valleys, environment is mostly mountainous yellow-brown desert soil	[42]
**5**	*F.syreitschikowii* K.-Pol.	Yili, Tacheng, Bole, Xinjiang	The environment is mostly low mountain brown calcareous soils, wastelands and gravelly slopes	[43]
**6**	*F. songarica* Pallas ex Sprengel	Tacheng and Altay, Xinjiang	The environment is mostly in mountainous grassy slopes and mountainous bushes	[42]
**7**	*F. krylovii* Korov.	Tori County, Xinjiang	The environment is mostly clayey saline grassland	[44]
**8**	*F. lehmannii Boiss.*	Manas County, Xinjiang	On low mountain slopes at 600~700 m elevation, the environment is mostly clayey gravelly sandy calcareous soil	[45]
**9**	*F. bungeana* Kitagawa	Heilongjiang, Jilin, Liaoning, Inner Mongolia, Hebei, Henan, etc.	The environment is mostly sandy and gravelly desert soil or near sandy areas	[46]
**10**	*F. dissecta* (Ledeb.) Ledeb	Tacheng Region, Altay Region, Junggar Basin, Xinjiang	The environment is mostly sandy and gravelly desert slopes, mostly in the mountains dominated by *Artemisia* spp.	[47]
**11**	*F. jaeschkeana* Vatka	Ali, Zada, Tibet	The environment is mostly at 3600 m above sea level on mountain slopes	[48]
**12**	*F. akitschkensis* B. Fedtsch. ex K.-Pol.	Xinjiang Alatau Mountains, Altai Mountains and western Junggar Mountains	The environment is mostly mountain shrublands and gravelly slopes at 900~2100 m above sea level.	[3]
**13**	*F. ovina* (Boiss.) Boiss.	Altai, Tacheng, Xinjiang	The environment is mostly gravelly hillsides	[3]
**14**	*F. hexiensis* K. M. Shen	Southern Gansu	The environment is mostly hillside with low humidity	[3]
**15**	*F. olivacea* (Diels) Wolff ex Hand.-Mazz.	Lijiang, Yunnan	The environment is mostly in canyons and mountain gaps, woods and grasses	[49]
**16**	*F. caspica* M. Bieb.	Burqin and Tacheng, Xinjiang	The environment is mostly low mountain slopes and mountain gaps and desert areas	[50]
**17**	*F. sumbul* (Kauffm.) Hook. f.	Zhaosu County, Xinjiang	The environment is mostly mountainous scrub and gravel slopes	[49]
**18**	*F. licentiana* Hand.-Mazz.	Taihang Mountains	The environment is mostly valley grassland at 400~600 m above sea level	[51]
**19**	*F. licentiana* Hand.-Mazz.	Jiangsu, Shandong, Anhui, etc.	The environment is mostly sunny slopes, mountain rock crevices and hillsides	[52]
**20**	*F. gracilis* (Ledeb.) Ledeb.	Xinjiang Altai Region	The environment is mostly meadows, riverside forest edges and gravelly mountain slopes	[3]
**21**	*F. karataviensis* (Regel et Schmaih.) Korov.	Xinyuan County, Xinjiang	The environment is mostly gravelly hillsides	[3]
**22**	*F. dubjanskyi* Korov. ex Pavlov	Gansu Province, Anxi and Su Bei, Mahaoshan area	The environment is mostly desert and Gobi desert in the sand and dunes	[53]
**23**	*F. canescens* (Ledeb.) Ledeb.	Fuyun County, Xinjiang	Gravelly hillsides in a mostly desert environment	[3]
**24**	*F. kingdon-wardii* Wolff	Northwest Yunnan	The environment is mostly grassy slopes and rock crevices at 2700~3200 m above sea level	[54]
**25**	*F. kirialovii* Pimenov	Tianshan Mountains and Junggar Basin, Xinjiang	The environment is mostly gravelly grassy slopes and shrubby places at an altitude of about 1500 m	[55]
**26**	*F. lapidosa* Korov.	Chabchal County, Xinjiang	The environment is mostly mountainous gravelly slopes and grassy areas	[3]

**Table 2:** Coumarin compounds in domestic *Ferula* plants.

No	Compound	Type	Literature
**1**	(3′*S*, 8′*R*, 9′*S*, 10′*R*)-sinkianol A	A	[52]
**2**	(5′*S*, 8′*R*, 9′*S*, 10′*R*)-ferukrinone	A	[52]
**3**	Ferukrin	A	[52]
**4**	(3′*S*, 5′*S*, 8′*R*, 9′*S*, 10′*R*)-kellerin	A	[52]
**5**	(3′*S*, 5′*S*, 8′*R*, 9′*S*, 10′*R*)-deacetylkellerin	A	[52]
**6**	Farnesiferol A	A	[52]
**7**	Farnesiferone A	A	[52]
**8**	Gummosin	A	[52]
**9**	Polyanthinin	A	[52]
**10**	(3′*R*, 5′*R*, 10′*R*)-sinkianol B	B	[52]
**11**	Farnesiferol B	B	[52]
**12**	Farnesiferol C	B	[52]
**13**	Galbanic acid	B	[52]
**14**	Methyl galbanate	B	[52]
**15**	Sinkiangenol A	A	[52]
**16**	Karatavicinol	C	[52]
**17**	Umbelliprenin	C	[52]
**18**	Sinkiangenol B	A	[53]
**19**	Sinkiangenol C	B	[53]
**20**	Sinkiangenol D	A	[53]
**21**	Sinkiangenol E	A	[53]
**22**	Sinkiangenol F	E	[53]
**23**	2,3-Dihydro-7-hydroxy-2R*,3S*-dimethyl-2- [4-methyl-5-(4-methyl-2-furanyl)-3(*E*)-pentenyl]-furano[3,2-*c*] coumarin	D	[53]
**24**	2,3-Dihydro-7-hydroxy-2R*,3R*-dimethyl-2- [4-methyl-5-(4-methyl-2-furanyl)-3(*E*)-pentenyl]-furano[3,2-*c*]coumarin	D	[53]
**25**	12′-hydroxy-karatavicinol	C	[53]
**26**	Fekrynol	B	[53]
**27**	Actylfekrynol	B	[53]
**28**	Ferocaulidin	A	[53]
**29**	Fekrol	B	[53]
**30**	Ferucrinone	A	[53]
**31**	Deacetylkellerin	A	[53]
**32**	Kellerin	A	[53]
**33**	Colladocin	A	[53]
**34**	Lehmannolol	A	[53]
**35**	Kamolone	A	[53]
**36**	Assafoetidnol B	A	[53]
**37**	Assafoetidnol A	A	[53]
**38**	Lehmannolone A	A	[54]
**39**	Assafoetidin	B	[54]
**40**	Lehmannolone	B	[55]
**41**	Sinkianone	B	[55]
**42**	Colladonin	E	[56]
**43**	Episamarcandin	A	[57]
**44**	Sinkiangenorin D	A	[57]
**45**	Fekolone	B	[57]
**46**	Feselol	A	[57]
**47**	Ferulin A	D	[58]
**48**	Ferulin B	D	[58]
**49**	Ferulin C	D	[58]
**50**	2,3-Dihydro-7-hydroxy-2S*,3R*-dimethyl-2-[4,8-dimethyl-3(*E*),7-nonadienyl]-furo[3,2-*c*] coumarin	D	[58]
**51**	2,3-Dihydro-7-hydroxy-2R*,3R*-dimethyl-2-[4,8-dimethyl-3(*E*),7-nonadienyl]-furo[3,2-*c*] coumarin (DAW22)	D	[58]
**52**	2,3-Dihydro-7-hydroxy-2S*,3R*-dimethyl-2-[4-methyl-5-(4-methyl-2-furanyl)-3(*E*)-pentenyl]-furo-[3,2-*c*] coumarin	D	[58]
**53**	2,3-Dihydro-7-hydroxy-2R*,3R*-dimethyl-2-[4-methyl-5-(4-methyl-2-furanyl)-3(*E*)-pentenyl]-furo[3,2-*c*] coumarin	D	[58]
**54**	2,3-Dihydro-7-methoxy-2S*,3R*-dimethyl-2-[4,8-dimethyl-3(*E*),7-nonadienyl]-furo[3,2-c] coumarin	D	[58]
**55**	2,3-Dihydro-7-methoxy-2R*,3R*-dimethyl-2-[4,8-dimethyl-3(*E*),7-nonadienyl]-furo[3,2-*c*] coumarin	D	[58]
**56**	2,3-Dihydro-7-hydroxy-2S*,3R*-dimethyl-3-[4,8-dimethyl-3(*E*),7-nonadienyl]-furo[3,2-c] coumarin	D	[58]
**57**	2,3-Dihydro-7-hydroxy-2R*,3R*-dimethyl-3-[4,8-dimethyl-3(*E*),7-nonadienyl]-furo[3,2-*c*] coumarin	D	[58]
**58**	4,7-Dihydroxy-3-[3,7,11-trimethyl-2(E),6(E),10-dodecatrienyl] coumarin	E	[58]
**59**	2,3-Dihydro-7-hydroxy-2R*,3R*-dimethyl-2-[4,8-dimethyl-3(*E*),7-nonadiene-6-onyl] furo[3,2-*c*] coumarin	D	[59]
**60**	Fukanefuromarin A	D	[59]
**61**	Fukanefuromarin B	D	[59]
**62**	Fukanefuromarin C	D	[59]
**63**	Fukanefuromarin D	D	[59]
**64**	Fukanemarin A	E	[59]
**65**	Fukanefuromarin H	D	[60]
**66**	Fukanefuromarin I	D	[60]
**67**	Fukanefuromarin J	D	[60]
**68**	Fukanefuromarin K	D	[60]
**69**	Fukanefuromarin L	D	[60]
**70**	Fukanefuromarin M	E	[60]

**Table 3:** Sesquiterpenoids in Domestic *Ferula* Plants.

No	Compound	Literature
**71**	Ferulaeone A	[61]
**72**	Ferulaeone B	[61]
**73**	Ferulaeone C	[61]
**74**	Ferulaeone D	[61]
**75**	Ferulaeone E	[61]
**76**	Ferulaeone F	[61]
**77**	Ferulaeone G	[61]
**78**	Ferulaeone H	[61]
**79**	Dshamirone	[61]
**80**	(4*E*,8*E*)-1-(2,4-dihydroxyphenyl)-2-hydroxy-5,9,13-trimethylt-etra-deca-4,8,12-trien-1-one	[61]
**81**	(6*E*)-1-(2,4-dihydroxyphenyl)-3,7,11-trimethyl-3-vinyl-6,10-dodecadien-1-one	[61]
**82**	(6*E*)-1-(2,4-dihydroxyphenyl)-3,7-dimethyl-3-vi-nyl-8-(4-methyl-2-furyl)-6-octen-1-one	[61]
**83**	3-(2,4-dihydroxybenzoyl)-4S*,5R*-dimethyl-5- [4,8-dimethyl-3(*E*),7(*E*)-nonadien-1-yl] tetrahydro-2-furanone	[61]
**84**	3-(2-hydroxyl-4-methoxybenzoyl)-4S*,5R*- dimethyl-5- [4,8-dimethyl-3(*E*),7(*E*)-nonadien-1-yl] tetrahydro-2-furanone	[61]
**85**	3-(2,4-dihydroxybenzoyl)-4R*,5R*-dimethy-5- [4,8-dimethyl-3(*E*),7(*E*)-nonadien-1-yl] tetrahydro-2-furanone	[61]
**86**	1-(2′,4′-dihydroxyphenyl)-3,7,11-trimethyl-3-vinyl-6(*E*),10-dodecadien-1-one	[62]
**87**	2,3-dihydro-7-hydroxy-2,3-dimethyl-2-[4′,8′-dimethyl-3′,7′-nonadienyl] -furo [3,2,c] coumarin	[62]
**88**	2,3-dihydro-7-hydroxy-2,3-dimethyl-3-[4′,8′-dimethyl-3′,7′-nonadienyl] -furo [3,2,c] coumarin	[62]
**89**	Fukanefurochromones A	[63]
**90**	Fukanefurochromones B	[63]
**91**	Fukanefurochromones C	[63]
**92**	Fukanefurochromones D	[63]
**93**	Fukanefurochromones E	[63]
**94**	2,3-dihydro-7-hydroxy-2S*,3R*-dimethyl-2-[4,8-dimethyl-3(*E*),7-nonadienyl]-furo[2,3-b] chromone	[63]
**95**	Fukanedone A	[64]
**96**	Fukanedone B	[64]
**97**	Fukanedone C	[64]
**98**	Fukanedone D	[64]
**99**	Fukanedone E	[64]
**100**	Fukaneketoester A	[64]
**101**	Feruone	[5]
**102**	5*α*-(*p*-hydroxybenzyl) ester of ferutriol	[65]
**103**	Ferutriol	[65]
**104**	5*ɑ*-isovalerate of ferutriol	[65]
**105**	Jaeschkeanadiol	[65]
**106**	Lapidin	[65]

**Table 4:** The composition of volatile oils of *F. sinkiangensis*, *F. fukanensis*, *F. ferulaeoides*, *F. ovina*.

No	Compound	Molecular Formula	Literature
**107**	(1*S*)-*β*-Pinene	C_10_H_16_	[73]
**108**	1,7,7-Trimethyl-tricyclo[2.2.1.0 (2,6)] heptane	C_10_H_16_	[73]
**109**	(1*R*)-*α*-Pinene	C_10_H_16_	[73]
**110**	Camphene	C_10_H_16_	[73]
**111**	6-Methyl-5-hepten-2-one	C_8_H_14_O	[73]
**112**	*β*-Pinene	C_10_H_16_	[73]
**113**	*α*-Phellandrene	C_10_H_16_	[73]
**114**	3-Carene	C_10_H_16_	[73]
**115**	Terpinolene	C_10_H_16_	[73]
**116**	1-Isopropyl-2-methylbenzene	C_10_H_16_	[73]
**117**	d-Limonene	C_10_H_16_	[73]
**118**	(*E*)-3,7-Dimethyl-1,3,6-octatriene	C_10_H_16_	[73]
**119**	(*Z*)-3,7-Dimethy-1,3, 6-octatriene	C_10_H_16_	[73]
**120**	*γ*-Terpinene	C_10_H_16_	[73]
**121**	l-Camphor	C_10_H_16_O	[73]
**122**	Borneol	C_10_H_16_O	[73]
**123**	(−)-α-Copaene	C_15_H_24_	[73]
**124**	2-Methoxy-4-methyl-1-(1-methylethy)-benzene	C_11_H_16_O_2_	[73]
**125**	1-Methoxy-4-methyl-2-(1-methylethyl)-benzene	C_11_H_16_O	[73]
**126**	*L*-Bornyl acetate	C_12_H_20_O_2_	[73]
**127**	Lavandulol acetate	C_12_H_20_O_2_	[73]
**128**	(+)-α-Longipinene	C_15_H_24_	[73]
**129**	*(Z)*-2,6,10-Trimethy-1,5,9-undecatriene	C_14_H_24_	[73]
**130**	Caryophyllene	C_15_H_24_	[73]
**131**	(*1S*,*2S*,*4R*)-2-Acetate-1,3,3-trimethyl -bicyclo[2.2.1] hepten-2-ol	C_12_H_20_O_2_	[73]
**132**	1-Ethenyl-1-methyl-2-(1-methylethenyl)- 4-(1-methylethylidene)-cyclohexane	C_15_H_24_	[73]
**133**	*1S*-(1α,4α,7α)]-1,2,3,4,5,6,7,8-Octahydro -1,4-dimethyl-7-(1-methylethylenyl)-azule	C_15_H_24_	[73]
**134**	(4αS,9αR)-2,4α,5,6,7,8,9,9α-Octahydro- 3,5,5-trimethyl-9-methyllene-benzocycloheptene	C_15_H_24_	[73]
**135**	(3Z,6*E*) -3,7,11-Trimethyl-1,3,6,10-dodecanetetraene	C_15_H_24_	[73]
**136**	*α*-Farnesene	C_15_H_24_	[73]
**137**	Isolongjflene-8-ol	C_15_H_26_O	[73]
**138**	Tran-nerolidol	C_15_H_26_O	[73]
**139**	Guaiol	C_15_H_26_O	[73]
**140**	*α*-Eudesmol	C_15_H_26_O	[73]
**141**	(2*R*,4*αR*)-1,2,3,4,4α,5,6,7-Octanhydro-α, α -3,8-tetramethyl-naphthalenemethanol	C_15_H_26_O	[73]
**142**	[3*S*-(3*α*,3*aβ*,5*α*)]-1,2,3,3*α*,4,5,6,7-Octahydro-α, α -3,8-tetramethyl-5-azulenemethanol	C_15_H_26_O	[73]
**143**	(1*S*)-1-[(1*S*)1,5-Dimethyl-4-hexen-l- 4-methyl-3-cyclohexen-1-ol	C_15_H_26_O	[73]
**144**	Isoledene	C_15_H_24_	[73]
**145**	Ethanol	C_2_H_6_O	[74]
**146**	Ethyl ester	C_4_H_8_O_2_	[74]
**147**	3-Methyl-butanal	C_5_H_10_O	[74]
**148**	2-Butanethiol	C_4_H_10_S	[74]
**149**	2,5-Dimethyl-furan	C_6_H_8_O	[74]
**150**	Furfural	C_5_H_4_O_2_	[74]
**151**	Methyl 1-propenyl disulfde	C_4_H_8_S_2_	[74]
**152**	Dimethyl trisulfide	C_2_H_6_S_3_	[74]
**153**	*n*-Propyl *sec*-butyl disulfide	C_7_H_16_S_2_	[74]
**154**	Propyl *n*-butyl disulfide	C_7_H_16_S_2_	[74]
**155**	1,2-Dithiacyclopentane	C_3_H_6_O_2_	[74]
**156**	2-Ethyl-hexanethiol	C_8_H_18_S	[74]
**157**	*bis* (1-Methylpropyl)-disulfide	C8H18S2	[74]
**158**	2,2′-Bioxirane	C_4_H_6_O_2_	[74]
**159**	3-Methyl-4-heptanol	C_8_H_18_O	[74]
**160**	1,1-*bis* (Methylthio)-ethane	C_4_H_10_O_2_	[74]
**161**	1,1-Dimethoxy-propane	C_5_H_12_O_2_	[74]
**162**	Heptadecane	C_17_H_36_	[74]
**163**	2-Ethylthio-butane	C_6_H_14_S	[74]
**164**	α-Humulene	C_15_H_24_	[74]
**165**	β-Selinene	C_15_H_/_	[74]
**166**	Hexadecane	C_16_H_32_	[74]
**167**	2-Methylthio butyrat methyl ester	C_6_H_12_OS	[74]
**168**	2,3-Dimeth yl-3-hexanol	C_8_H_18_O	[74]
**169**	Hedycaryol	C_15_H_26_O	[74]
**170**	2,2-*bis* (Methylthio)-propane	C_5_H_12_S_2_	[74]
**171**	Octadecatriene	C_18_H_32_	[74]
**172**	Palmitic acid	C_16_H_32_O_2_	[74]
**173**	Oleic acid	C_18_H_34_O_2_	[74]
**174**	*α*-Myrcene	C_10_H_16_	[75]
**175**	*o*-Cymene	C_10_H_14_	[75]
**176**	Enol	C_10_H_16_O	[75]
**177**	Limonene oxide	C_10_H_16_O	[75]
**178**	Camphor	C_10_H_16_O	[75]
**179**	Thymol	C_10_H_14_O	[75]
**180**	*α*-Terpineol	C_10_H_18_O	[75]
**181**	Anisyl acetate	C_12_H_2_O_2_	[75]
**182**	Carvacrol acetate	C_10_H_16_O	[75]
**183**	1-Methoxy-4-methyl-2-(1-ethyl)-benzene	C_11_H_16_O	[75]
**184**	Carvone	C_10_H_14_O	[75]
**185**	Bornyl acetate	C_12_H_2_O_2_	[75]
**186**	(−)-Verbenone	C_10_H_16_O	[75]
**187**	*α*-(*E*)-BETA-FARNESENE	C_15_H_24_	[75]
**188**	1-Cycloethyl-1-pentyne	C_11_H_18_	[75]
**189**	*α*-Bergamotene	C_15_H_24_	[75]
**190**	1,11-Hexadecadiyne	C_16_H_26_	[75]
**191**	*α*-Bisabolene	C_15_H_24_	[75]
**192**	Asarone	C_12_H_16_O_3_	[75]
**193**	3,7,11-trimethyl(*E*)1,6,10-dodecatrien-3-ol	C_15_H_26_O	[75]
**194**	Farnesol	C_15_H_26_O	[75]
**195**	Graphene oxide	C_15_H_24_O	[75]
**196**	Disulfide bis(1-methylpropyl)	C_8_H_18_S_2_	[75]
**197**	*n*-Propyl sec-butyl disulfide	C_7_H_16_S_2_	[76]
**198**	Ocimene	C_10_H_16_	[76]
**199**	Ocimene (Mixture of isomers)	C_10_H_16_	[76]
**200**	Disulfide, bis[1-(methylthio)ethyl]	C_6_H_14_S_4_	[76]
**201**	2-Methylbutyl benzene	C_11_H_16_	[76]
**202**	Ethyl 1-methylpropyl disulfide	C_6_H_14_S_2_	[76]
**203**	(*Z*)-1,6,10-Dodecatriene-7,11-dimethyl-3-methylene	C_15_H_24_	[76]
**204**	cis-α-Bisabolene	C_15_H_24_	[76]
**205**	Dipropyl disulfide	C_6_H_14_S_2_	[76]
**206**	Hinesol	C_15_H_26_O	[76]
**207**	Neoisolongifolene	C_15_H_22_	[76]
**208**	Bicyclo[4.4.0]dec-l-ene,2-isopropyl-5-methyl-9-methylene	C_15_H_24_	[76]
**209**	2*H*-Pyran, tetrahydro-4-methyl-2-(2-methyl-1-propenyl)	C_10_H_18_O	[76]
**210**	2,4,6-Octatriene, 2,6-dimethyl-, (*E*,*Z*)-	C_10_H_16_	[76]
**211**	Estragole	C_10_H_12_O	[76]
**212**	1-Methoxy-4-methyl-2-(1-methylethyl) Benzene	C_11_H_16_O	[76]
**213**	2,6-Dimethyl-2,6-octadiene	C_10_H_18_	[76]
**214**	Methyl 2-(methylthio)butyrate	C_6_H_12_O_2_S	[76]
**215**	1-Methyl-4-(1-methylethyl)-1,3-cyclohexadiene,	C_10_H_16_	[76]
**216**	Methyl sec-butyl disulphide	C_6_H_12_O_2_S	[76]
**217**	3-(Methylthio)-2-butanone	C_5_H_10_OS	[76]
**218**	Naphthalene,1,2,3,4.4a,5,6,8a-octahydro-7 -methyl-4-methylene-1-(1-methyl-ethyl)-, (1α.,4aβ.,8aα)-	C_15_H_22_	[76]
**219**	1,4-Methanoazulene, decahydro-4,8,8-trimethyl-9-methylene-, [IS-(l.a.,3a.β.,4a.,8a.β)]-	C_15_H_26_O	[76]
**220**	Tetrahydro thiazole	C_5_H_12_S_2_	[76]
**221**	(4a*S*-*cis*)-2,4a,5,6,7,8,9,9a-octahydro- 3,5,5-trimethyl-9-methylene -1H-Benzocycloheptene	C_5_H_10_OS	[76]
**222**	Thiopropionamide	C_3_H_7_NS	[76]
**223**	Longifolene-(V4)	C_15_H_24_	[76]
**224**	Naphthalene, 1,2,3,4,4a,5,6,8a-octahydro -4a,8-dimethyl-2-(1-methyl ethenyl)-, [2R-(2α.,4aα.,8aβ)]	C_15_H_24_	[76]
**225**	Seychellene	C_3_H_7_NS	[76]
**226**	(*R*)-2,4a,5,6,7,8-hexahydro-3,5,5,9-tetramethyl-1*H*-benzocycloheptene	C_15_H_24_	[76]
**227**	[*S-(Z)*]-3,7,11-trimethyl-1,6,10-Dodecatrien-3-ol	C_15_H_24_	[76]
**228**	*E*-Famesene epoxide	C_3_H_7_NS	[76]
**229**	Di-epi-α-cedrene	C_15_H_24_	[76]

## Data Availability

Not applicable.
